# Thermal preferences and limits of *Triatoma brasiliensis* in its natural environment - Field observations while host searching

**DOI:** 10.1590/0074-02760150234

**Published:** 2015-09

**Authors:** Silvia Catalá, Claudia Mendonça Bezerra, Lileia Diotaiuti

**Affiliations:** 1 Consejo Nacional de Investigaciones Científicas y Técnicas, Centro Regional de Investigaciones Científicas y Transferencia Tecnológica de La Rioja, La Rioja, Argentina; 2 Secretaria da Saúde do Estado do Ceará, Núcleo de Controle de Endemias, Fortaleza, CE, Brasil; 3 Fundação Oswaldo Cruz, Centro de Pesquisas René Rachou, Belo Horizonte, MG, Brasil

**Keywords:** Triatominae, Chagas disease, thermal limits

## Abstract

The goal of this work was to explore the thermal relationship between foraging* Triatoma brasiliensis* and its natural habitat during the hottest season in the state of Ceará, Brazil. The thermal profiles were determined using infrared analysis. Although the daily temperature of rock surfaces varied in a wide range,* T. brasiliensis *selected to walk through areas with temperatures between 31.7-40.5ºC. The temperature of* T. brasiliensis *body surface ranged from 32.8-34.4ºC, being higher in legs than the abdomen. A strong relationship was found between the temperature of the insect and the temperature of rock crevices where they were hidden (r: 0.96, p < 0.05). The species was active at full sunlight being a clear example of how the light-dark rhythm may be altered, even under predation risk. Our results strongly suggest a thermal borderline for* T. brasiliensis *foraging activity near 40ºC. The simultaneous determination of insect body and rock temperatures here presented are the only obtained in natural habitats for this or other triatomines.

*Triatoma brasiliensis* (Hemiptera: Reduviidae: Triatominae) now represents the most significant insect vector of *Trypanosoma cruzi* - causative agent of Chagas disease - in the northeastern *Caatinga* region of Brasil (Silveira et al. 2001, Costa et al. 2003, 2014). But, although susceptible to available insecticides, domestic populations of *T. brasiliensis* can be difficult to control because treated premises can be easily re-infested from sylvatic ecotopes. Four months after spraying with deltamethrin, 9.7% of the houses were still positive, especially the peridomiciliary ecotopes (Diotaiuti et al. 2000). The persistence of *T. brasiliensis *in the artificial environments after the traditional chemical control is due to the density of this species in the wild and to the fact that human habitation is, in a way, inside or very close to the wild habitats (Silveira et al. 2001).

In the wild, *T. brasiliensis* is mainly found amongst the exposed rock pile, typical habitats of the hot dry *Caatinga* landscape, often in association with rodents, marsupials or bats (Alencar 1987, Bezerra et al. 2014). In rock-free sedimentary lowlands, it can be occasionally associated to the cactus*Pilosocereus gounellei* (Valença-Barbosa et al. 2014). The temperature and humidity profiles of the rock crevices tend to be similar to those of the local domestic habitats (Lorenzo et al. 2000). Laboratory experiments under controlled humidity and temperature showed that *T. brasiliensis*modified its thermal preferences when starving, moving from 29-26ºC with increasing starvation (Guarneri et al. 2003).

The critical temperatures (minimum and maximum) within which the individuals are generally active contribute to determine the thermal tolerance of the species and its physiological niche. This and other Triatominae species modulate their thermal preference according to their physiological state. The rate of variation in preferred temperatures is altered when the insects are starved (Lazzari 1991, Schilman 1998, Pires et al. 2002). Moreover, active dispersion of triatomine bugs is triggered by starvation and it is being more frequent during the hot season (Schofield et al. 1991, 1992,Abrahan et al. 2011). Therefore, the more critical season for the insects is the most risky period for the invasion and colonisation of domestic habitats.

The *Caatinga* ecoregion, home to 15 million people, is a semiarid region with only two distinguishable seasons: the very hot and dry and the hot and rainy. During the peak periods of drought (August-January) (Vasconcellos et al. 2010) the soil can reach temperatures of up to 60ºC and the vegetation and fauna of the region manage to survive in this environment. Even being a well adapted species, *T. brasiliensis *is not an exception, but how much risk does the insect take not to starve in this extremely hot environment?

The goal of this work was to explore *in nature* the thermal relationship between foraging *T. brasiliensis *and its natural habitat under critical survival conditions. This was not an experimental work; researchers (as observers) acted as a feeding stimuli in the natural habitat of *T. brasiliensis*.

## MATERIALS AND METHODS

This study was carried out in October 2010 in the rural area of the municipality of Tauá, state of Ceará, northeastern Brazil. The average annual temperature varies between 26-28ºC, with an average rainfall of 597.2 mm^3^ and a rainy season from February-April. October mean temperature is 26.4ºC (21.6-31.2ºC). The driest months are September and October. A more detailed description of the study area can be found in Bezerra et al. (2014).

A typical stony area near Cachoeira do Julio (Bezerra et al. 2014) was selected to observe the behaviour of *T. brasiliensis *in its natural wild habitat. This area corresponds to a point of Bezerra et al. (2014) study where rodents have high occurrence.

*Thermography of the active T. brasiliensis and its natural habitat* - A Forward Looking Infrared (FLIR) i40 infrared (IR) camera wavelength range of 7-14 µm, accuracy of ± 2% and thermal sensitivity < 0.1ºC at 25ºC (FLIRi40.com), was used to obtain thermal images (IR resolution 14,400 pixels 120 x 120) of the habitats of *T. brasiliensis* during two consecutive days (18-19 October 2010) from 09:00-11:00 am and 02:00-10:00 pm.

Environmental monitoring using FLIR cameras determine the temperature of objects and landscapes through the detection of IR radiation typically emitted from a heat source. Thermographic cameras are very good instruments to obtain precise and direct measurement of temperature differences of identical objects, but not among objects of different nature. The reason is that the camera only detects IR radiation, which varies not only with the temperature of an object, but also with the material its emitting surface is composed of. In order to compare temperatures among objects of different nature (stones, insect cuticle) the coefficient of emissivity of different materials was corrected in the thermal images. This *a posteriori*correction allowed us to get the correct temperature value for each frame when those images were analysed with the QuickReport software (FLIR user manual, FLIR Systems, 2007). Emissivity was fixed at 0.98 for insect cuticle (Lahondère & Lazzari 2012) and 0.81 for rocks (granite, FLIR user manual, FLIR Systems, 2007).

The insect’s surfaces were scanned with the FLIR camera while they walked over the rocks, responding to the human presence. The temperature of rock crevices (craks, caves) where the insects were hidden was determined as the temperature of the basal rock on the hollow.

*Statistical analysis - *The following variables were determined in the images: minimum and maximum temperature of the rocks surfaces, minimum and maximum temperature of rock crevices, minimum and maximum *T. brasiliensis* body surface temperature and minimum and maximum temperature of the walking surface (around the insect´s body). The median, range and quartiles of these variables were calculated using STATISTICA 2000 (StatSoft). A lineal relationship between the temperature (ºC) of the rock crevices and the temperature of the *T. brasiliensis* body when emerging from these shelters was determined by regression analysis.

## RESULTS

*The temperature on rocks* - Even on the same rock, the temperature varied from area to area according to insolation, the surrounding vegetation and the rock constitution. The surface temperatures during the observation periods ranged from 27.6ºC (absolute minimum) to 68.5ºC (absolute maximum). Table shows the median, quartiles and minimum/maximum values of rocks temperature. The highest thermal amplitude on the stones surface (33.4ºC) was registered at midday (35.1-68.5ºC).

**TABLE:** Median, range and quartiles of rock temperature on surface and caves were *Triatoma brasiliensis* live in Tauá, state of Ceará, Brazil, median, range and quartiles of the insect temperature while foraging over the rocks

	n	Median (ºC)	Range (ºC)		Quartile (ºC)	
					Lower Upper	
Rock minimum	59	35.9	27.6	48	33.6	37.8
Rock maximum	59	37.9	33.2	68.5	36.6	42.8
Cave minimum	37	33.5	28	38.5	32	34.8
Cave maximum	37	36.6	30.2	42.2	33.6	37.7
*T. brasiliensis *minimum	38	32.8	28	35.5	32.4	34.9
*T. brasiliensis* maximum	38	34.4	30.1	37.1	33.7	35.9

Within the rock crevices, a less pronounced temperature variation was observed. The medians of minimum and maximum temperatures in these cracks were 33.5ºC and 36.6ºC respectively (Table).

*T. brasiliensis behaviour and body temperature* - During the late afternoon, early evening and early morning, our proximity to the rocks in the natural habitat attracted 11 *T. brasiliensis: *six fifth instar nymphs, four adults and one third stage nymph. No insects appeared during the hottest hours, from 09:30 am-06:00 pm. All the insects had flat abdomens, characteristic of a fasting status, and were very active and motivated to feed. They came out from rock cracks, ran directly to us and spontaneously extended their proboscis when a hand was offered. A female flew from the rock to the shoulder of one of the researchers.

**Fig. 1:**
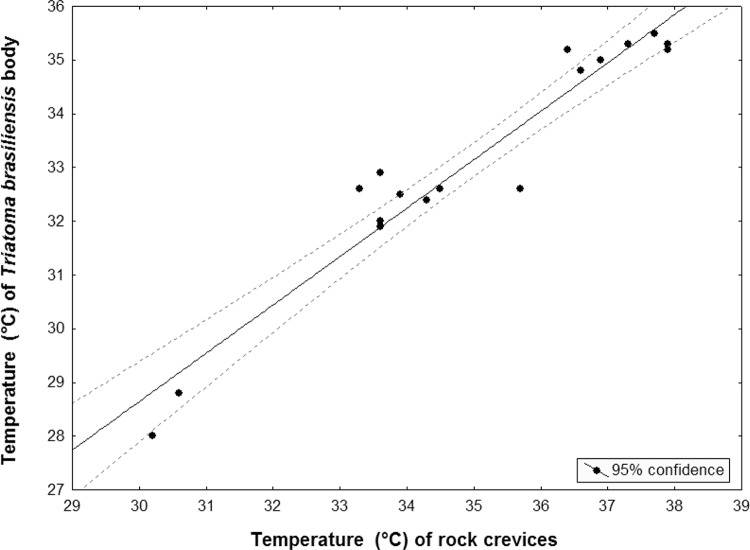
: relationship between the temperature of the rock crevices and the temperature of the *Triatoma brasiliensis* body when emerging from these shelters (n = 17, r: 0.96, p < 0.05).

The temperature of *T. brasiliensis* body surface (Table) ranged from a median of 32.8-34.4ºC, being higher in legs than the abdomen/thorax. Body maximum and minimum temperatures were higher in the late afternoon. A strong relationship was found between the temperature of the insect body and the temperature of the rock crevices where *T. brasiliensis* was hidden (Fig. 1) (n = 17, r: 0.96, p < 0.05).

**Fig. 2:**
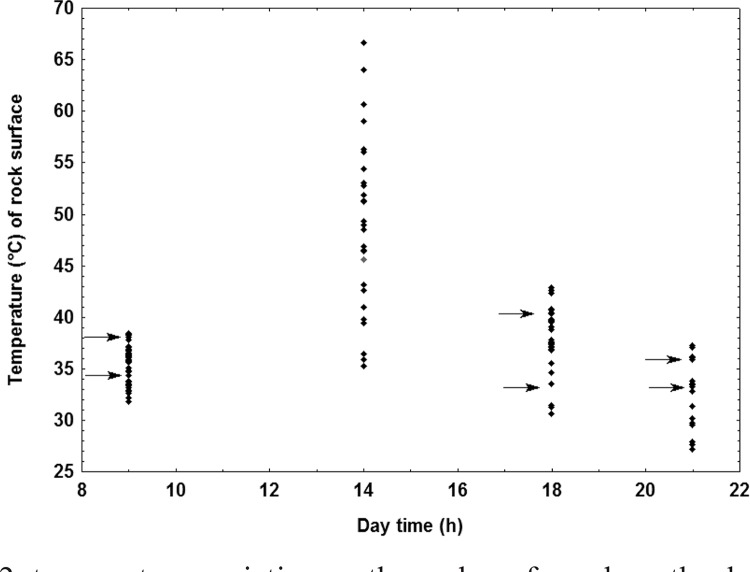
: temperature variation on the rock surface along the day and temperature range of the walking surface selected by *Triatoma brasiliensis *(arrows)*.*

In general, while moving towards the human host, the insects ran over surfaces under 39ºC and, extraordinarily, at higher values (walking surface temperature range: 31.7-40.5ºC). During late afternoon the insects faced the hottest surfaces to walk on (Fig. 2).

## DISCUSSION

The preferred temperature range is an essential characteristic of species that defines their ability to reach and colonise new habitats as well as its geographic distribution. The thermotolerance range is a critical trait that determines the physiological niche of each species (Spicer & Gaston 1999) and it could be delimited by critical temperatures (minimum and maximum) within which the individuals are generally active (Chown & Nicolson 2004). Studies concerning modelled geographical distribution and thermotolerance of Chagas disease vectors are mainly referred to *Triatoma infestans *and *Rhodnius prolixus,* the most important vectors of *T. cruzi*(de la Vega et al. 2015). Laboratory experiments on thermal preferences of many triatomine species are performed with laboratory insects reared under particular fixed conditions. The results of our work point out the utility of field observations and measurements in natural scenarios in order to know the real limits of the species. Our data, even limited in time, show that hungry *T. brasiliensis* walks over hot substrates. However, the thermotolerance and thermopreference of the species will need a deeper analysis to be established.

Triatominae species show marked temporal organisation in their behaviour. They are inactive during the day inside their refuges and become active at first hours of night, searching for food, mating opportunities and oviposition sites (Guerenstein & Lazzari 2009). According toBezerra et al. (2014), *T. brasiliensis* at all developmental stages left their hiding places after darkness and walked over the stone surfaces returning to their hideouts a few hours later (10:00-11:00 pm). As expected, we also registered *T. brasiliensis* nymphs and adults foraging early at night, the most adequate period in terms of favourable rocks temperature and maximum possibility for IR detection of host (Catalá 2011). Surprisingly, they were active looking for a host at full sunlight (e.g., 09:30 am) and even before sunset (e.g., 06:00 pm), corroborating the aggressive behaviour of this species increased by the prolonged starvation. This is a clear example of how, under certain circumstances, the light-dark rhythm may be altered, even when predation risk increases.

Despite the fact that the temperature of the rock surfaces varied in a wide range (= 40.9ºC) during the day, *T. brasiliensis* selected to walk through areas with temperatures between 31.7-40.5ºC, being absent at midday when the rocks reached a maximum of 68.5ºC (Fig. 2). These data strongly suggest a thermal borderline for *T. brasi-liensis*foraging activity near 40ºC and stress the adaptive capacity of the species to survive in this environment.

The thermal analyses of the infested stone crevices showed a more gentle environment, with minimum and maximum medians of 33.5ºC and 36.6ºC, respectively, a little variation in magnitude when compared with the broader oscillations of the external environment. The ectothermic condition of insects was evidenced by the high relationship of the insect body temperature and the temperature of these bug shelters (Fig. 1).

This is the first time that the body temperature range is registered for a Triatominae species under natural conditions. We noted that the highest values were recorded in the legs while the abdomen was colder, supporting the idea that insects of this species adopt a characteristic stance, maintaining the body at a great distance from the ground as a strategy to avoid harmful temperatures (Guarneri et al. 2003).

Andrew et al. (2013) suggest that the ant*Iridomirmex purpureus* may be using behavioural cues - e.g., raising their gaster - to deal with the extreme temperatures when foraging in extreme heat conditions. The ant is able to perform at a great running capacity with no sign of speed reduction. This author calls into question the general application of thermal performance curves to predict likely extinction risk, without taking into account behavioural flexibility.

On the other hand, triatomine species can exhibit extreme thermal sensitivity which allows them to distinguish heat sources differing only a few hundredths of a degree (Lazzari & Nuñez 1989). Guerenstein and Lazzari (2009) and Schmitz et al. (2000) demonstrated that the thermal stimulation alone can induce the approach and extension of the proboscis when searching for food. Our observations of *T. brasiliensis*approaching a hand may support this mechanism but, at these short distances, we must also consider the importance of smells emanating from the host and the multimodal integration of external cues (Lazzari et al. 2013).

This paper shows that, under natural extreme conditions, hungry *T. brasiliensis *challenge temperatures at the highest limit, even at diurnal light, in order to get food. The recorded temperatures from habitat and insects surpass the previous values on thermal preference obtained from this species in laboratory experiments (Lorenzo et al. 2000).
